# The *ENA1* Na^+^-ATPase Gene Is Regulated by the SPS Sensing Pathway and the Stp1/Stp2 Transcription Factors

**DOI:** 10.3390/ijms24065548

**Published:** 2023-03-14

**Authors:** Abdelghani Zekhnini, Marcel Albacar, Antonio Casamayor, Joaquín Ariño

**Affiliations:** Institut de Biotecnologia i Biomedicina, Departament de Bioquímica i Biologia Molecular, Universitat Autònoma de Barcelona, 08193 Cerdanyola del Vallès, Spain; abdelghani.zekhnini@uab.cat (A.Z.); marcel.albacar@uab.cat (M.A.); antonio.casamayor@uab.cat (A.C.)

**Keywords:** transcriptional regulation, environmental alkalinization, stress response, *Saccharomyces cerevisiae*

## Abstract

The *Saccharomyces cerevisiae ENA1* gene, encoding a Na^+^-ATPase, responds transcriptionally to the alkalinization of the medium by means of a network of signals that involves the Rim101, the Snf1 and PKA kinases, and the calcineurin/Crz1 pathways. We show here that the *ENA1* promoter also contains a consensus sequence, located at nt −553/−544, for the Stp1/2 transcription factors, the downstream components of the amino acid sensing SPS pathway. Mutation of this sequence or deletion of either *STP1* or *STP2* decreases the activity of a reporter containing this region in response to alkalinization as well as to changes in the amino acid composition in the medium. Expression driven from the entire *ENA1* promoter was affected with similar potency by the deletion of *PTR3*, *SSY5*, or simultaneous deletion of *STP1* and *STP2* when cells were exposed to alkaline pH or moderate salt stress. However, it was not altered by the deletion of *SSY1*, encoding the amino acid sensor. In fact, functional mapping of the *ENA1* promoter reveals a region spanning from nt −742 to −577 that enhances transcription, specifically in the absence of Ssy1. We also found that the basal and alkaline pH-induced expression from the *HXT2*, *TRX2*, and, particularly, *SIT1* promoters was notably decreased in an *stp1 stp2* deletion mutant, whereas the *PHO84* and *PHO89* gene reporters were unaffected. Our findings add a further layer of complexity to the regulation of *ENA1* and suggest that the SPS pathway might participate in the regulation of a subset of alkali-inducible genes.

## 1. Introduction

Adaptation to changes in the environment is critical for cell survival. The yeast *Saccharomyces cerevisiae* grows preferentially at acidic pH, and even a moderate alkalinization of the extracellular pH triggers an adaptive response that is largely based on the remodeling of gene expression affecting hundreds of genes. Work performed in the last 20 years has delineated in *S. cerevisiae* a number of signaling pathways that are responsible for such response, including the Rim101, Pho4, Slt2/Rlm1, Snf1 and PKA kinases, and calcineurin/Crz1 pathways [1,2].

The *S. cerevisiae ENA1* gene encodes a P-type ATPase able to export Na^+^ and K^+^ cations that become the only way to eliminate toxic Na^+^ cations in the absence of extracellular/intracellular H^+^ gradient. Under standard growth conditions, *ENA1* is barely expressed, but its promoter is potently induced shortly after cells are shifted to a medium containing high levels of Na^+^ or under alkaline pH [3,4,5,6,7]. The mechanisms for gene induction are various. Under mild saline (0.3–0.4 M NaCl) stress, *ENA1* induction is predominantly due to the activation of the HOG pathway [8], which acts by phosphorylating the bZip transcription factor Sko1 [9] and recruiting the histone deacetylase complex Rpd3-Sin3 to the *ENA1* promoter to facilitate transcriptional activation [10]. Induction of *ENA1* by higher concentrations of Na^+^ also involves the activation of calcineurin [8], which dephosphorylates the transcription factor Crz1 leading to its entry into the nucleus [11,12] and is binding to two calcineurin-dependent response elements (CDRE) in the *ENA1* promoter [13].

As mentioned above, *ENA1* is also potently induced by alkaline pH, and this effect is mediated by several mechanisms. One of them is the activation of calcineurin, promoted by a very fast influx of calcium cations into the cell [14]. The impact of the Ppz1 protein phosphatase on *ENA1* expression seems to be also mediated by the regulation of calcineurin [15]. A second mechanism consists of the activation of the Rim101 transcription factor, a conserved pathway in fungi. Activation of Rim101 releases Nrg1-mediated repression of the *ENA1* promoter, leading to transcriptional activation of the gene [16,17]. A third mechanism involves activation of the Snf1 kinase, which phosphorylates both Mig1 and Mig2 repressors, and also acts by releasing Nrg1-mediated repression of *ENA1* [18]. Induction of *ENA1* expression by high pH does not involve the HOG pathway nor the PKA-regulated Msn2/Msn4 transcription factors [14,19], and the conjunction of the three mechanisms described above was considered sufficient to account for the transcriptional control of *ENA1* in response to environmental alkalinization [19]. Whereas the benefits of the induction of *ENA1* in response to salt stress seem obvious, those derived from its activation by alkaline pH were less evident. This was clarified after the finding that the signaling network controlling the induction of *PHO89* (encoding a high-affinity Na^+^/Pi cotransporter) in response to high pH was virtually identical to that identified for *ENA1* [20]. At alkaline pH, Pho89 becomes a key component for phosphate uptake, but this is achieved at the expense of the import of Na^+^ cations. Therefore, concomitant induction of *ENA1* would counteract the accumulation of Na^+^, thus allowing maintenance of the extracellular/intracellular cation gradient required to sustain Pho89-mediated Pi uptake and/or prevent the intracellular accumulation of Na^+^ to toxic levels.

Bioavailable nitrogen is indispensable for life, and yeast cells growing in different environments are exposed to a variety of nitrogen sources. Therefore, the sensing of such sources becomes essential for these cells in order to reprogram their metabolism for optimal proliferation [21]. Sensing of extracellular amino acids is made possible by the SPS sensor system. This system is composed of three proteins located at the plasma membrane: Ssy1, Ptr3, and Ssy5. Ssy1 displays high similarity to several amino acid permeases, but it does not transport amino acids: instead, it has a sensor function provided by its N-terminal region [22,23,24,25]. Ssy1 interacts with Ptr3 and Ssy5 to form the complete sensor system that is stimulated by extracellular amino acids. The signal is transmitted through Ptr3 and promotes the interaction with the protein kinases Yck1/2, which phosphorylates Ptr3 and Ssy5 [26]. Consequently, Ssy5 is partially proteolyzed, releasing its endoprotease activity. Activated Ssy5 then acts on the transcription factors Stp1 and Stp2 by removing its N-terminal negative regulatory domains and promoting their translocation into the nucleus and the activation of their target genes [27,28,29,30].

During the course of our research on the generation of hybrid promoters able to respond to alkalinization, we incorporated to alkaline-responsive synthetic reporters short DNA segments from the *ENA1* promoter containing the known Nrg1 or Mig1/2 consensus sequences with the aim of down-regulating their basal expression. Surprisingly, whereas the Nrg1-containing fragment acted as expected, the one harboring the Mig1/2 sequence further activated expression. In this work, we demonstrate that this anomalous behavior is due to the existence of an Stp1/Stp2 consensus sequence adjacent to the Mig1 site. We also show that the elimination of components of the SPS pathway, but not that of the Ssy1 sensor, attenuates expression from the entire *ENA1* promoter. These findings highlight a novel and unexpected regulatory input for the control of *ENA1* expression and possibly other alkaline pH-responsive genes.

## 2. Results

### 2.1. A Short Region of the ENA1 Promoter Containing the Mig1/2 Consensus Sequence Unexpectedly Increases the Activity of a Hybrid Promoter

**Figure 1:**
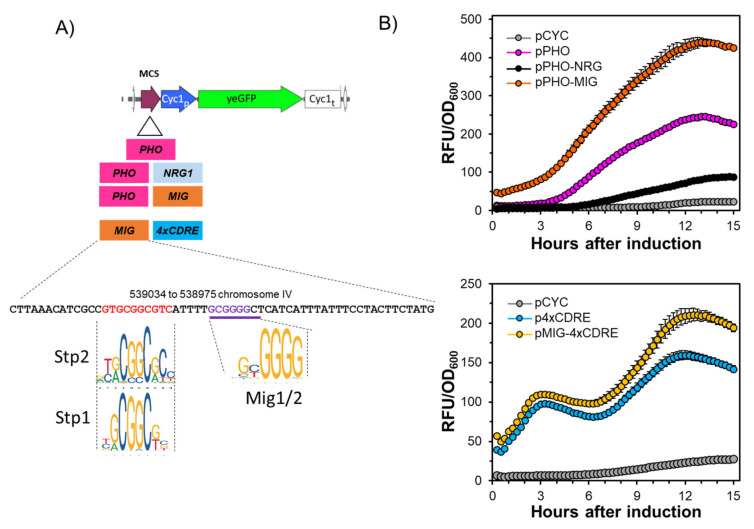
A 60-bp fragment of the *ENA1* promoter that contains the Mig1/2 site potentiates transcription. (**A**) Schematic depiction of the diverse constructs driving yeGFP expression tested in panel B. At the bottom, the relevant section of the *ENA1* promoter (with its chromosomal coordinates) shows the previously identified Mig1/2 site and the predicted Stp1/2 consensus sequence. (**B**) BY4741 cultures transformed with the indicated reporters were grown as indicated in Material and Methods, and the fluorescence and growth (OD_600_) values were captured after raising the pH to 7.9–8.0 every 15 min for 15 h. Data are expressed for each culture as relative fluorescence units (RFU) divided by OD_600_ values and are presented as the mean ± SEM from 6 (upper panel) or 4 (lower panel) independent cultures.

With the aim of testing hybrid promoters likely able to respond to alkaline pH, we developed a set of constructs composed of a basal *CYC1* transcription sequence fused to eGFP and containing upstream consensus sequences for different alkaline sensitive transcription factors. Figure 1A shows the structure of one of these constructs, in which a 37 nt fragment containing two Pho4-binding sites from the *PHO84* gene was placed upstream of the *CYC1* basal transcription region. As shown in Figure 1B (upper panel), the inclusion of the Pho4 elements (pPHO) promoted sustained expression of the GFP reporter after the alkalinization of the medium. In order to test the effect of negative elements, we placed downstream of the Pho4 elements an Nrg1 consensus sequence (55 nt) derived from the −552/−606 region of the *ENA1* promoter (Figure 1A).

As expected, the insertion of this sequence markedly decreased the levels of GFP. We next introduced (also downstream of the *PHO* element) a 60 nt region of the *ENA1* promoter (−508/−567) known to contain a functional Mig1/2 repressor element (from now on denoted as MIG). However, to our surprise, the addition of this region did not repress the expression of the reporter but, on the contrary, resulted in a marked increase in GFP levels (Figure 1B, upper panel). To test further this unexpected behavior, we placed the MIG fragment upstream of a region containing a cluster of four CDRE elements (4xCDRE) that was previously known to respond to high pH [14]. As shown in Figure 1B (lower panel), the inclusion of the MIG fragment also increased the expression from this promoter.

The results described above suggested that the MIG fragment contained a positive transcriptional element not yet identified. Indeed, computational analysis of this region revealed that slightly upstream of the previously characterized Mig1/2 site, there was a region showing high identity with an Stp2 consensus sequence (*p*-value 1.8 × 10^−5^). Stp2 and its paralog Stp1 (with a very similar consensus sequence, see Figure 1A) are transcription factors activated by proteolysis in response to external amino acids and are known to activate the transcription of genes coding for diverse amino acid permeases.

### 2.2. The Putative Stp1/2 Site Present in the ENA1 Promoter Is Relevant for Transcriptional Induction

**Figure 2:**
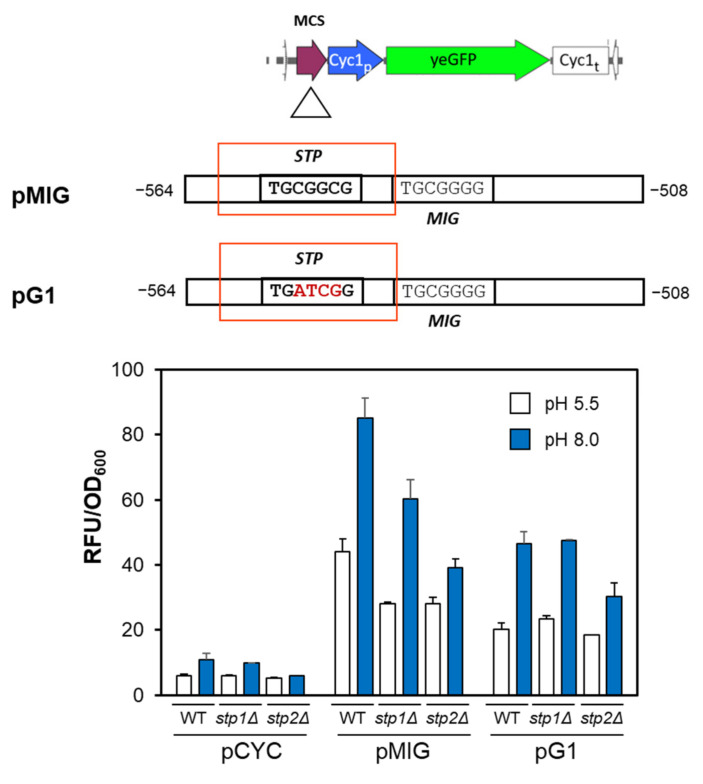
Mutation of the putative Stp1/2 site decreases expression from the pMIG reporter. Upper panel. The structure of the MIG region was cloned into the pCYC vector. The nucleotides mutated in pG1 are denoted in red. The lower panel shows the expression at normal or alkaline pH from the empty vector (pCYC), pMIG, or pG1 in BY4741 wild-type cells (WT) or its *stp1*Δ and *stp2*Δ derivatives. In this case, induction was carried out for 6 h, cells were fixed for flow cytometry, and fluorescence was recorded with the fluorimeter. Data are mean ± SEM from three independent experiments.

The transcriptional relevance of the putative Stp1/2 recognition sequence was investigated by cloning in the basal *CYC1* reporter vector the native MIG fragment alone as well as a mutated version in which the nearly constant CGGC central residues were changed to ATCG (pG1, see Figure 2). Both constructs were then introduced in BY4741 wild-type cells, as well as in *stp1* and *stp2* deletion mutants. As shown in Figure 2, fragment MIG alone was able to enhance transcription from the *CYC1* basal promoter in wild-type cells growing in standard conditions, and the effect was increased further in cells switched to alkaline pH. The expression of GFP upon alkalinization was reduced in cells lacking Stp1 and particularly in those lacking Stp2. The effect of the mutation at the Stp1/2 site (pG1) was also noticeable, yielding expression levels comparable to those observed in the *stp2* deletion mutant for the native pMIG reporter. These results indicate that the MIG fragment of the *ENA1* promoter contains a *bona fide* Stp1/2 consensus sequence that participates in the induction prompted by alkaline pH. They also suggest that Stp2 might be more important than Stp1 for regulation at this site.

**Figure 3:**
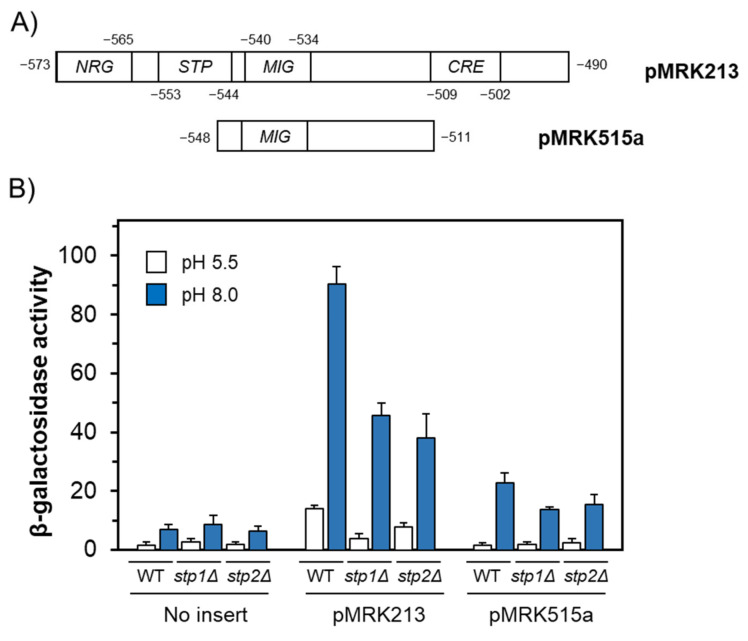
Removal of the putative Stp1/2 site drastically decreases expression from a *lacZ* reporter. (**A**) Schematic depiction of the constructs used. (**B**) Strain BY4741 (WT) and its isogenic *stp1* and *stp2* derivatives were transformed with the indicated constructs or the empty pSLFΔ-178K vector (“No insert”). Cells were treated as indicated in Materials and Methods and β-galactosidase activity was determined after 1 h of induction. Data are mean ± SEM from 4 to 6 independent experiments.

To confirm the relevance of the newly detected Stp1/2 site, we resorted to alternative *ENA1*-derived β-galactosidase reporters (Figure 3A) previously characterized in our laboratory for alkaline response [19]. As shown in Figure 3B, the presence of the −573/−490 region of the *ENA1* promoter (reporter pMRK213) conferred a pH-dependent response to the reporter in wild-type cells, and this response was substantially decreased when the reporter was introduced in *stp1* or *stp2* deletion mutants. This region comprises the Nrg1/2, Stp1/2, and Mig1/2 sites, as well as a CRE (cyclic AMP response element) element, albeit the latter has no relevance in alkaline pH transcriptional regulation [19]. When the same experiment was repeated using the pMRK515a construct, which only contains the Mig1/2 site, alkaline pH induction in wild-type cells was much lower (~four-fold) and was barely affected by the mutation of the *STP1* or *STP2* genes. These experiments indicate that the Stp1/2 site identified here could be relevant for the regulation of the induction of the *ENA1* ATPase gene in response to environmental alkalinization.

### 2.3. The Stp1/2 Site in the ENA1 Promoter Responds to the Activation of the SPS Pathway

**Figure 4:**
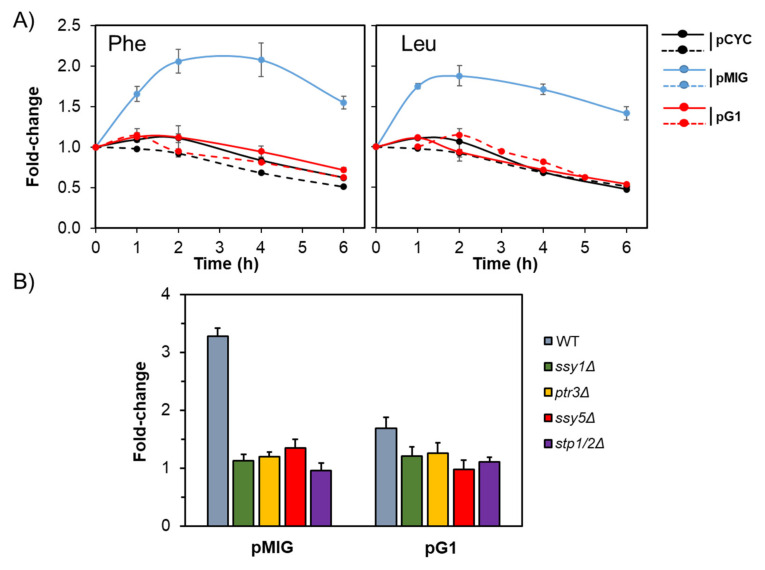
The Stp1/2 site in the *ENA1* promoter responds to amino acids. (**A**) Strain CEN.PK113-5D was transformed with the indicated constructs and was grown in a minimal medium with Pro as the only nitrogen source, as described in Materials and Methods. Then, 1.5 mM Leu or Phe was added (solid lines), and growth continued. Samples were taken at the indicated periods of time and processed for flow cytometry. (**B**) Amino acid-induced response of the Stp1/2 site is abolished in mutants in the SPS pathway. The indicated strains (23344c background) carrying the pMIG or pG1 constructs were switched from Pro to Phe-containing medium, and samples were processed for flow cytometry after 2 h. In all cases, the ratio of RFU values of the induced vs non-induced cultures was calculated and is expressed as the mean ± SEM from six independent experiments.

As mentioned in the introduction, Stp1 and Stp2 transcription factors are known to be activated through the SPS pathway, which responds to amino acid availability. We then wondered whether the Stp1/Stp2 site present in the *ENA1* promoter could be sensitive to amino acid signals. To test this possibility, we introduced the basal pCYC1 reporter, as well as the pMIG and pG1 versions in the strain CEN.PK113-5D, which is prototrophic for amino acids and auxotrophic only for uracil. Cells were grown in a synthetic medium with Pro as the only N source (a condition known to maintain inactive the SPS pathway) and then switched to a medium with Leu or Phe to activate the SPS pathway. As shown in Figure 4A, the pMIG reporter was rapidly induced by changing Pro by either Leu or Phe, whereas this effect was completely abolished in cells bearing the pG1reporter, in which the Stp1/2 site had been mutated. This observation indicated that the predicted Stp1/2 element in the *ENA1* promoter was responsive to the activation of the SPS pathway. To explore in more detail the involvement of the SPS pathway in this response, we resorted to the 23344c background, a Σ1278b-based strain in which mutations in the pathway are available and have been widely characterized. As shown in Figure 4B, all these mutations blocked the response of the pMIG reporter when cells were shifted from Pro to Phe, similarly to that observed for the pG1 reporter.

**Figure 5:**
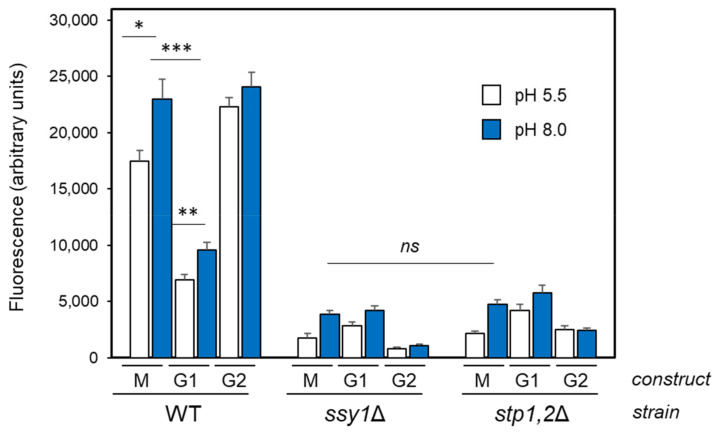
Expression of pMIG (M), pG1, and pG2 in the 23344c background. The indicated reporters (pG2 bears a mutated Mig1/2 site) were introduced into strains 23344c (WT), 32501c (*ssy1*Δ), or KW023 (*stp1*Δ *stp2*Δ). Cells were exposed to alkaline pH for 2 h and processed for flow cytometry analysis. Data are the mean ± SEM from 8 to 10 independent experiments. *ns*, not significative, *p* > 0.05; *, *p* < 0.05; **, *p* < 0.01; ***, *p* < 0.005.

We also tested the response of the pMIG and pG1 reporters in the 23344c genetic background upon alkalinization and incorporated a new reporter (pG2) in which the Mig1/2 site was changed from 5′-TGC**GGG**G-3′ to 5′-TGC**ATC**G-3′. As shown in Figure 5, in spite of the rather high basal activity of the pMIG reporter in this genetic background, it was further enhanced by alkalinization. Removal of the Stp1/2 site clearly decreased both basal and alkaline pH-induced expression levels, whereas elimination of the Mig1/2 consensus increased basal expression to levels similar to those of alkali-induced cells, which was compatible with the elimination of a repressor site. The expression levels from all three constructs were drastically decreased in cells lacking both Stp1 and Stp2 transcription factors or devoid of the Ssy1 sensor. Taken together, these results indicate that the newly found Stp1/2 site responds to the activation of the SPS pathway.

### 2.4. Non-Homogenous Impact of Mutations in the SPS Pathway on the Expression Driven from the Entire ENA1 Promoter

**Figure 6:**
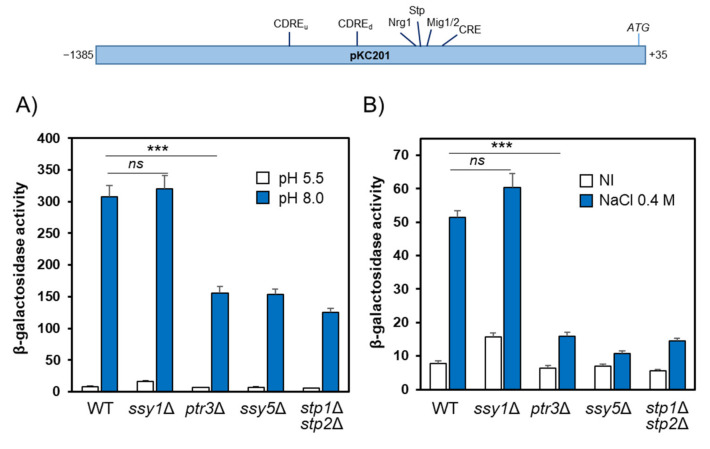
Effect of mutations in the SPS pathway on the expression of the full *ENA1* promoter. Plasmid pKC201, bearing the entire *ENA1* promoter fused to *lacZ*, was introduced into strain 23344c (WT) and its isogenic *ptr3*, *ssy5*, *ssy1*, and *stp1* *stp2* derivatives. Cells were exposed for 1 h to alkaline pH (**A**) or 0.4 M NaCl (**B**) and processed for β-galactosidase activity determination. “NI” denotes not induced. Data are mean ± SEM from 4 to 10 independent experiments. ***, *p* < 0.005, *ns*, not significant. The gene structure and relevant regulatory sites in the *ENA1* promoter included in plasmid pKC201 are indicated in the cartoon shown at the top. CDREu and CDREd refer to the upstream and downstream elements, respectively.

To investigate the potential effect of the activation of the SPS pathway on the expression of the *ENA1* gene, we resorted to the pKC201 reporter, which is a *LacZ* translational fusion containing the entire *ENA1* promoter region, and introduced this construct into the wild-type BY4741 strain and its *stp1*Δ and *stp*2Δ derivatives. As shown in Figure S1, in this genetic background, *ENA1* was potently induced by alkalinization, and mutation of either *STP1* or *STP2* significantly decreased its expression. This suggests that both transcription factors might operate on the *ENA1* gene. When the expression of the entire *ENA1* reporter was analyzed in the 23344c background, simultaneous removal of both Stp1 and Stp2 transcription factors resulted in a marked decrease of the expression, and a similar effect was observed in cells lacking the Ptr3 and Ssy5 components of the signaling pathway (Figure 6A). However, to our surprise, the deletion of the gene encoding the Ssy1 sensor did not affect expression in response to alkaline pH. Because *ENA1* also responds to moderate salt stress, we treated the cells with 0.4 M NaCl. As shown in Figure 6B, this response strongly decreased in cells lacking both Stp1 and Stp2 transcription factors, as well as in the *ptr3*Δ and *ssy5*Δ mutants. Remarkably, again the activity of the *ENA1* reporter did not decrease in cells devoid of the Ssy1 sensor. This was a very surprising result since it indicated that the alkaline pH- and NaCl-induced upregulation of the *ENA1* promoter depends on the presence of elements of the SPS pathway but not on the presence of the Ssy1 sensor.

### 2.5. Functional Mapping of the Alkaline pH Response of the ENA1 Promoter and Its Relationship with the SPS Pathway

**Figure 7:**
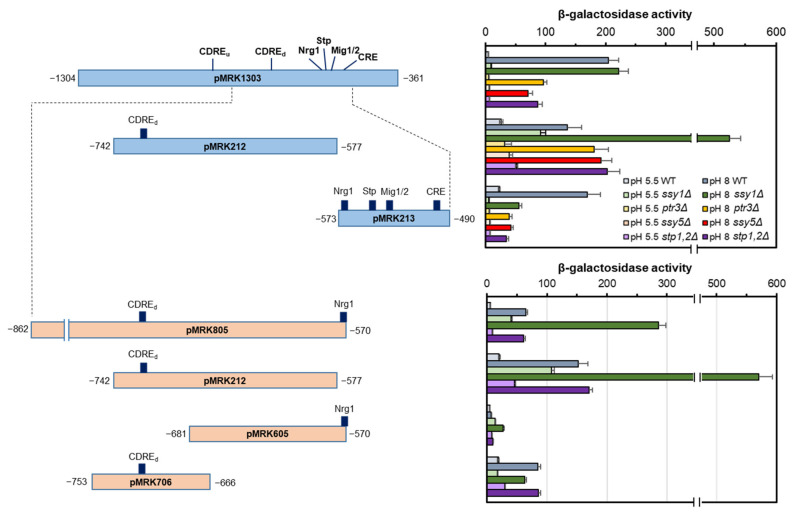
Functional mapping of the alkaline pH response of the *ENA1* promoter in mutants in the SPS pathway. The diverse strains (23344c background) were transformed with pSLFΔ-178K derived reporters containing the indicated fragments, and cells were exposed for 1 h to alkaline pH prior to determination of β-galactosidase activity. The relevant regulatory sites included in each fragment are depicted. Data are mean ± SEM from 6 to 10 independent experiments. A second set of independent determinations for pMRK212 is included in the bottom section for better comparison with the newly constructed reporters.

Because of the unexpected behavior of the *ENA1* promoter in cells lacking Ssy1, we aimed to identify the region of the promoter responsible for this behavior. To this end, we cloned diverse regions of the promoter in plasmid pSLFΔ-178K (as in Figure 3), and tested the response to high pH in strains lacking diverse components of the SPS signaling pathway. As presented in Figure 7 (upper panel), the expression profile from plasmid pMRK1303 perfectly mimicked that observed for plasmid pKC201, suggesting that it contains all elements required for response to high pH. Expression from plasmid pMRK213 was induced by high pH (as observed for strain BY4741, Figure 3) and it was clearly reduced, in a similar way, in all SPS mutants, including the *ssy1* strain. This induction could be attributable to the combination of a depression effect, attributable to the Mig1/2 site and insensitive to the deletion of *SSY1*, and the activation due to the Stp1/2 site, fully blocked by the *ssy1* mutant (compare plasmids pMRK506 and pMRK515a in Figure S2). In contrast, expression from plasmid pMRK212 showed an interesting pattern. Basal levels were somewhat higher than with pMRK213, and this effect was particularly marked in the *ssy1* mutant. When cells were challenged with high pH, expression was similar to that observed in the wild-type strain for all mutants, with the exception of the *ssy1* strain, in which β-galactosidase activity was two- to three-fold higher.

From this experiment, it was evident that the promoter region responsible for the abnormal behavior of *ENA1* expression in *ssy1* cells could be restricted to the fragment cloned in plasmid pMRK212, delimited by positions −742/−577. To further limit the anomalous region, we constructed three additional plasmids and tested them in wild-type, *ssy1,* and *stp1 stp2* cells. pMRK805 contained the pMRK212 region plus about 120 nt upstream. As shown in Figure 7 (lower panel), expression from this region is still markedly enhanced in the *ssy1* strain but not in *stp1 stp2* cells. Plasmid pMRK706 contains a fragment of less than 90 bp that includes the downstream CDRE motif located at −726/−718. In this case, expression is increased by high pH in all three strains in a similar way, indicating that this region is not responsible for the atypical effect due to the *ssy1* mutation. Finally, the fragment in pMRK605, comprising the −681/−565 promoter region, does not respond at all to alkalinization in the wild-type and *stp1 stp2* strain, although it shows an increase of about three- to four-fold compared with the other strains, in the *ssy1* mutant. Therefore, the extraordinary behavior of the *ENA1* promoter in response to high pH in *ssy1* cells is not mediated by the functional CDRE and can be attributed to structural determinant(s) located between positions −681/−565.

### 2.6. Participation of Stp1/2 in the Expression of Other Genes Responsive to Alkaline pH

**Figure 8:**
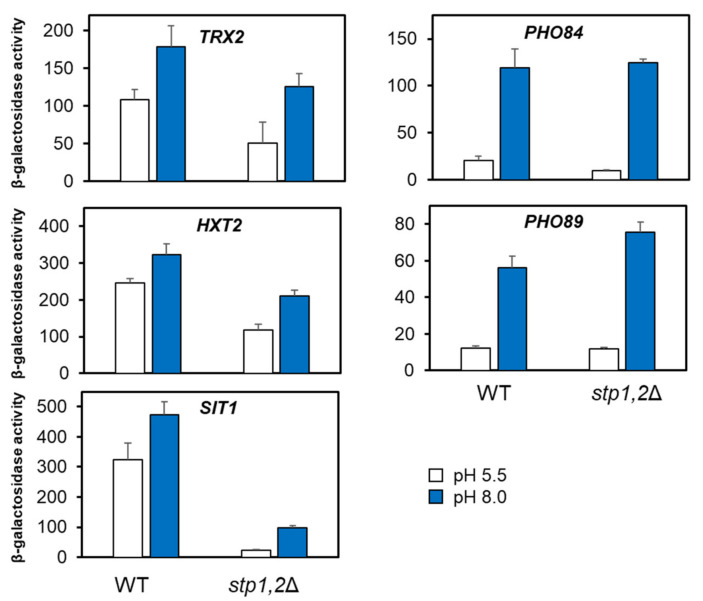
Effect of the lack of Stp1 and Stp2 transcription factors on the expression of diverse alkali-induced gene reporters. The indicated reporters were introduced in strain 23344c (WT) and KW023 (*stp1*Δ *stp2*Δ) and grown as in Figure 6. LacZ activity was measured after 1 h of induction at alkaline pH. Data are mean ± SEM from 4 to 10 independent experiments.

We then wondered whether the participation of the SPS pathway could be observed in other gene promoters also responsive to alkaline pH. To this end, we tested previously characterized *PHO84*-, *PHO89*-, *TRX2*-, *SIT1*-, and *HXT2*-*LacZ*-based reporters in the wild-type 23344c strain and its *stp1 stp2* derivative. As shown in Figure 8, the induction triggered by alkaline pH observed with *PHO89* and *PHO84* reporters did not decrease when these were tested in the *stp1 stp2* strain. For *TRX2*-, *HXT2*-, and *SIT1*-based reporters, the induction by alkaline pH was modest, but this was likely the result of abnormally high basal expression levels (see the Discussion section). In any case, both basal and pH-induced expression levels decreased moderately in the *stp1 stp2* strain for the *TRX2* and *HXT2* reporters, whereas they strongly diminished in the case of *SIT1*. To further confirm these results, we tested the *HXT2*-based reporter in the BY4741 background. As shown in Figure S3, in this genetic background, induction of the *HXT2* reporter was more robust and, whereas the deletion of *STP2* did not significantly alter expression levels, that of *STP1* resulted in around a decrease of 25%. This observation further supports the notion that *HXT2* might be under the control of the Stp1 transcription factor. Taken together, these results suggest that alkaline pH-responsive genes other than *ENA1* could be subjected to regulation by the transcription factors acting downstream of the SPS pathway.

## 3. Discussion

The results presented here indicate that the *ENA1* promoter contains a previously unidentified sequence that is recognized by the Stp1/2 transcription factors and is relevant for response to the alkalinization of the medium. This sequence is located at position −553/−544 and is able not only to enhance the expression driven from other alkali-responsive transcriptional elements but also capable by itself of increasing the expression of a basal *CYC1* promoter in response to alkalinization of the medium. In addition, this element responds to the shift from Pro as the sole N source to Phe or Leu, a situation that is known to trigger signaling through the SPS pathway [31], and the response entirely depends on the integrity of this pathway. This would suggest that alkalinization might activate the SPS pathway, a notion that would agree with the increased expression upon alkalinization observed for several genes, such as *AGP1*, *BAP2*, *DIP5,* or *MUP1* [18,32,33,34,35,36] that are also a target for the SPS pathway [37].

*ENA1* is a key factor for tolerance to salt and alkaline pH stress and responds to the activation of multiple pathways in response to various signals, including nutrient availability [38,39]. Among these signals, early work identified the relevance of the carbon source. Thus, the expression of *ENA1* is repressed by glucose in comparison with galactose [40], and this is mediated by activation of the Snf1-Mig1/2 axis [41]. Our observations indicate that *ENA1* also has the potential to respond to the activation of the SPS pathway, which senses external amino acids. Indeed, we show that the expression of *ENA1* in response to both alkaline pH and moderate salt stress (Figure 6) is reduced with similar potency by deletion of *PTR3*, *SSY5*, or simultaneous elimination of both *STP1* and *STP2* genes. To our surprise, deletion of the gene encoding the actual amino acid sensor, *SSY1*, was without effect under both kinds of stress when the entire promoter was tested. Functional mapping of the *ENA1* promoter carried out in the diverse SPS mutants showed that the region from nt −742 to −490 of the promoter, which integrates most of the alkaline pH signals [14,19], can be divided into two segments with very different behavior as far as the components of the SPS pathway are concerned. The region spanning from nt −573 to −490, which contains the known pH-sensitive Nrg1 and Mig1/2 sites, as well as the Stp1/2 site identified in this work, appears as markedly SPS-dependent in its response to high pH, showing a homogenous behavior for all mutations in the pathway. In contrast, the response of the adjacent upstream region (from nt −742 to −577) is unaltered in *ptr3*, *ssy5*, or *stp1 stp2* cells, but it is clearly enhanced in cells devoid of Ssy1. Remarkably, the region from −753 to −667 promoted a high pH response, and it was unaffected by the lack of Ssy1 and its downstream Stp1 and Stp2 transcription factors. This region contains the *ENA1* downstream CDRE, which is known to bind Crz1 with high affinity and constitutes the main determinant of the induction response to calcium and alkaline pH [13,42,43]. On the other hand, the region covering from nt −681 to −565 (pMRK605) was insensitive to alkalinization in the wild type, as previously shown [14] and in the *stp1 stp2* strain, but showed a significant increase (about four-fold) in the Ssy1-deficient strain (Figure 7).

These findings lead us to two conclusions. From one side, the absence of effect of the *ssy1* mutation could be interpreted as the compensatory balance between a negative effect caused by lack of signaling at the Stp1/2 site found at the −573/−490 region and a positive one promoted by an unknown target likely placed between nt −681 and −565. On the other side, the normal response of the region, including the CDRE in the *ssy1* mutant, indicates that the lack of Ssy1 is not influencing the signaling through the Crz1 binding site. A major remaining question would be the nature of the signal leading to hyperactivation of the response in the *ssy1* mutant. One possible explanation would be that the absence of the sensor protein might upset the balance between the downstream components of the SPS pathway, thus affecting other pathways that might impinge on the *ENA1* promoter. It is worth noting that it has been reported that the expression of numerous genes, including amino acid permeases *CAN1* and *DIP5*, subjected to NCR (nitrogen catabolite repression) regulation, was affected by the *ssy1* mutation independently of the presence or absence of leucine (used to induce the SPS pathway) in the medium [44]. Thus, it might be speculated that, in the conditions in which our experiments were carried out (rich medium), the absence of the SPS sensor might result in the activation of genes otherwise subjected to NCR control. In fact, lack of Ssy1 results in increased expression of *GAT1*, encoding a GATA transcription factor whose expression is normally down-regulated in nitrogen-rich media. This scenario would fit with the previous observation by Crespo and coworkers [45] that *ENA1* expression was induced in cells treated with rapamycin (an inhibitor of the TOR pathway). In addition, mutation of *GLN3* and *GAT1*, encoding the positive NCR transcription factors known to act downstream TOR, resulted in reduced basal and salt-induced expression of *ENA1* in response to salt stress.

Therefore, in this scenario, the abnormal activation driven by the *ENA1* promoter region spanning from −681 and −565 would be explained by a release from the NCR control. Gln3 and Gat1 are called GATA factors because they bind to (A/T)GATA(A/G) sequences. Crespo and coworkers [45] already pointed to the presence of several such sequences in the *ENA1* promoter (although no attempts were made to map them functionally). Interestingly, one of these is located at positions −638/−633 and, therefore, is present in the two reporters tested here (pMRK212 and pMRK605) that exhibit abnormal induction in Ssy1-deficient cells. It is worth noting that a role for Gat1 in the regulation of *ENA1* in *Hansenula polymorpha* has been proposed in response to the nitrogen source [46]. However, in this case, Gat1 would be activated by Crz1, a mechanism that is not likely to occur in response to alkalinization in *S. cerevisiae*.

Our exploration of different pH-responsive reporters indicates that alkaline pH-triggered activation of the SPS pathway is not restricted to the *ENA1* gene, as a loss of response is also observed for *TRX2, HXT2*, and *SIT1* reporter genes when both *STP1* and *STP2* genes are deleted (Figure 8). It is worth mentioning that the basal expression levels of the *HXT2* and *SIT1* reporters in the wild-type strain 23344c are much higher than that observed in the past for the same reporters in other genetic backgrounds [15,47], thus leading only to a modest increase upon alkalinization. This is not unexpected since 23344c is a Σ strain, rather distant from BY4741, which derives from the S288C background, and there is plenty of evidence showing that relevant phenotypic traits (including gene essentiality) can depend on the specific yeast genetic background [48,49,50]. We also observe a dramatic decrease in *stp1 stp2* cells in the basal and pH-induced response of the *SIT1* gene reporter. This is interesting because *SIT1* was identified as the only transporter gene showing the same induction pattern as *AGP1*, *GNP1*, *BAP3*, *TAT1*, and *TAT2* (all five genes dependent on the SPS sensor pathway) when cells were shifted from ammonium to various amino acids [51]. This effect on *SIT1* appears also dependent on the presence of Ssy1 [44]. In fact, analysis of the promoter regions of these genes reveals putative Stp1/2 consensus sequence: *TRX2* (positions −371/−352, *p* = 5.5 × 10^−6^), *HXT2* (positions −261/−242, *p* = 4.9 × 10^−6^) and *SIT1* (−319/−300, *p* = 1.3 × 10^−7^). In contrast, we do not observe the loss of alkaline pH induction in the *stp1 stp2* mutant when the *PHO84* and *PHO89* reporter genes are tested (Figure 7). This is not surprising in the case of *PHO84*, as it is known that its response to alkalinization is based solely on the activation of the PHO pathway and the presence of the Pho4 transcription factor [14]. In contrast, *PHO89* and *ENA1* have been found to be co-regulated in response to high pH, likely due to the requirement for a Na^+^-extrusion system working at alkaline pH to counteract the activity of the Pho89-mediated Na^+^/Pi cotransport and such co-regulation is based in that both promoters integrate the same set of signals [20]. Therefore, our finding illustrates a differential trait in the response of the *ENA1* and *PHO89* promoters to environmental alkalinization.

## 4. Materials and Methods

### 4.1. Yeast Strains and Culture Media

Yeast cells were incubated at 28 °C in YP medium (1% yeast extract, 2% peptone) plus 2% glucose except otherwise stated, or synthetic medium (YNB without amino acids) lacking the appropriate selection requirements [52]. The strains used in this work are indicated in Table 1. Searches of gene promoters for putative transcription factor binding sites were performed at the RSAT site using the Matrix-Scan algorithm [53].

### 4.2. Recombinant DNA Techniques and Reporter Construction

*Escherichia coli* DH5α cells were employed as the plasmid DNA host and were grown at 37 °C in the LB medium (1% tryptone, 0.5% Yeast Extract, 0.5% NaCl) supplemented, when required, with 50 μg/mL ampicillin. Transformations of *E. coli* and standard recombinant DNA techniques were performed as described [56]. *S. cerevisiae* cells were transformed by the lithium acetate method [57].

Constructs using eGFP as a reporter protein were based on the pRS426 (*URA3*, 2µ) reported in [58]. In the first step, a 1002 bp fragment containing the yeast-enhanced GFP (yeGFP) sequence followed by the *CYC1* terminator, was amplified from the pUG35 vector (Güldener and Hegemann, unpublished data, GenBank: AF298787.1) with primers GFP-term Fw and GFP-term Rev to provide pGFP-CYCterm. Vector pCYC was made by amplification of 269 bp fragment, containing the basal *CYC1* promoter region, from the pAMS366 plasmid [11] with primers CYC1promoter5′ and CYC1promoter3′, digestion with XhoI-ClaI, and cloning into these same sites of vector pGFP-CYCterm. Plasmid pCYC was used for subsequence constructions, except for pCYC-4xCDRE, which was constructed by amplification of 404 bp fragment from pAMS366 (containing the *CYC1* sequence and a 4x tandem of the CDRE site present in the *FKS2* gene) with primers 4x-CDRE5′ and CYC1promoter3′ and cloning into pGFP-CYCterm upon XhoI digestion.

Insertion of a *PHO* element was performed as follows. The −404/−440 region downstream of the *PHO84* promoter, containing the consensus Pho4 elements C and D described in [59], was synthesized by overlapped extension using oligonucleotides Pho84-pho4binding3′ and Pho84-pho4binding5′. The resulting 53 bp fragment was digested with BamHI and EcoRI and cloned into these sites in pCYC to yield pCYC-PHO. Individual Nrg1 and Mig1/2 sites were taken from the *ENA1* promoter [19]. The Nrg1 element (position −651/−647) was recreated by overlapped extension with oligonucleotides 5′-NRG1 and 3′-NRG1, yielding a 65 bp fragment. A 74-bp fragment containing the Mig1/2 site located at position −544/−534 of *ENA1* was synthesized by the same method using oligonucleotides 5′-MIG1/2 and 3′-MIG1/2. Each fragment was digested with HindIII and EcoRI and cloned into the same sites of pCYC, pPHO, or pCYC-4xCDRE to yield pCYC-MIG, pPHO-NRG, pPHO-MIG, or pMIG-4xCDRE (Figure 1A). Mutation of the putative Stp1/2 site was made as follows. An overlapped extension reaction was set with primers Mig1/2_NOStp2-5′ and 3′-MIG1/2, and the product was digested with HindIII and EcoRI and cloned into pCYC to yield pG1. The Mig1/2 site was changed using the same strategy with primers 5′-NOMig1/2 and 3′-NOMig1/2 to produce pG2. Overlapped extension reactions were made starting in all cases by mixing two hundred pmol of each oligonucleotide, raising the temperature heating up to 95 °C for 2 min and allowing annealing at 65 °C for 15 s in the presence of Q5 High-fidelity DNA polymerase and Q5 buffer (New England Biolabs™, Ipswich, MA, USA).

The construction of the following *LacZ* fusion plasmids has been previously reported as follows. The pKC201 vector contains the *ENA1* promoter sequence from −1385 to +35 [60]; plasmid pTRX2 carries the corresponding −981/+11 region [61]; plasmid pHXT2 includes the −1/−618 region of the *HXT2* gene [62]; plasmid pSIT1 contains the −786/+52 *SIT1* region [15]. Plasmids pPHO84 and PHO89 (603 to +19 and −671 to +33 regions, respectively) were described earlier [14]. Construction of plasmids pMRK1303, pMRK213, and pMRK212 containing, respectively, the −1304/−361, −573/−490, and −742/−577 regions of the *ENA1* promoter was described earlier [14]. These constructs derive from the vector pSLFΔ-178K, a *CYC1* promoter-*LacZ* fusion from which the *CYC1* UAS elements have been deleted [63]. Plasmid pMRK515a, which contains the −548/−511 region of the *ENA1* promoter in the pSLFΔ-178K vector, was made by PCR amplification of the relevant fragment of the *ENA1* promoter with primers ena1_prom_5′_G and ena1_prom_3′_E, followed by digestion with SalI and XhoI and cloning into the XhoI site of pSLFΔ-178K. Plasmids pMRK805 (−861/−565), pMRK706 (−753/−666), and pMRK605 (−681/−565) were made by amplification of the relevant region (see Table S1), cloned into pBluescript at the PstI and XhoI sites, released by SmaI and XhoI digestion, and cloned into the same sites of pSLFΔ-178K. Plasmid pMRK506, containing the −574 to −541 region, was made by hybridizing the pair of primers 5′-ENA1-509 and 3′-ENA1-509, digestion with SmaI and XhoI, and cloning into pSLFΔ-178K. All oligonucleotides used in this work can be found in Table S1.

All constructs were verified by restriction mapping and DNA sequencing.

### 4.3. Fluorimetric Determination of yeGFP Production

Cells carrying the relevant reporters were grown overnight in a synthetic medium lacking uracil and then diluted to an OD600 of 0.25 in the same medium supplemented with TAPS (pH 5.5) and grown until an OD600 of 0.5–0.6. Then, 292.5 μL of culture were added onto 96 well plates and supplemented (ThermoFisher, #165305) with 7.5 µL of KOH from a 1 M stock to raise the pH 7.9–8.0 or with the same volume of 1 M KCl (control cells). Plates were transferred to a Varioskan LUX Multimode Microplate Reader (ThermoFisher™, Waltham, MA, USA), and growth resumed at 28 °C with intermittent shaking. Fluorescence (relative fluorescence units at 488 nm excitation/520 nm emission) and absorbance (600 nm) were simultaneously measured every 15 min using the SkanIt™ software (ThermoFisher™, Waltham, MA, USA). Normalization of the fluorescence values was conducted by dividing by the corresponding OD600 values after subtracting the fluorescence and absorbance blank values.

### 4.4. Flow Cytometry Analysis

For determination of alkaline pH induction, aliquots of cultures of BY4741 or 23344c cells transformed with the appropriate reporters were grown until an OD600 of 0.5–0.6 and were made 25 mM KCl (non-induced) or 25 mM KOH (raising the pH to 7.9–8.0). Samples (0.8 mL) were taken at time zero and after different periods and fixed with formaldehyde (2.7%) for 5 min. After various washes with cold PBS, cells were diluted 1:20 to 1:40 with PBS and analyzed in a FACSCanto (Becton & Dickinson Co., Franklin Lakes, NJ, USA) flow cytometer.

Monitoring the reporter’s response to amino acids was carried out as follows. A colony of the wild-type strains CEN.PK113-5D or 23344c (as well as its derivatives), transformed with the suitable plasmids, was grown in a minimal medium with 10 mM Pro as the only amino acid source to saturation and then inoculated into the same medium and grown until OD_600_ = 0.6. Samples were taken just before adding Phe or Leu at 1.5 mM (t = 0) and then after different periods of incubation and processed for flow cytometry as described above.

### 4.5. β-Galactosidase Assays

The activity of the *LacZ* reporters was determined as in [19], except that non-induced cells were resuspended in a YPD medium adjusted to pH 5.5 after autoclaving. For the *HXT2*-*LacZ* reporter, YP supplemented with 4% glucose was used, as described in [47]. Induction of the cells was extended for 60 min.

## Figures and Tables

**Table 1:** Yeast strains employed in this work.

Strain	Genotype	Reference
BY4741	*MAT*a *his3*Δ1 *leu2*Δ0 *met15*Δ0 *ura3*Δ0	[54]
BY*stp1*Δ	BY4741 *stp1*::kanMX4	[55]
BY*stp2*Δ	BY4741 *stp2*::kanMX4	[55]
CEN.PK113-5D	*MAT*a *ura3*-52 *TRP1 LEU2 HIS3 MAL2*-8c *SUC2*	Euroscarf
23344c	*MAT*α *ura3-52*	[25]
32501c	*MAT*a *ssy1*::kanMX2 *ura3*	[25]
FB98	*MAT*α *ptr3*::kanMX2 *ura3*	[23]
FB90	*MAT*α *ssy5*::kanMX2 *ura3*	[23]
KW023	*MAT*α *stp1*Δ *stp2*Δ *ura3*	[31]

## Data Availability

Data is contained within this article and its supplementary material. Raw (unprocessed) data are available on request from the corresponding author.

## Supplementary Materials

The following supporting information can be downloaded at: https://www.mdpi.com/article/10.3390/ijms24065548/s1.
