# Supplementary material for: The ENA1 Na+-ATPase Gene Is Regulated by the SPS Sensing Pathway and the Stp1/Stp2 Transcription Factors
Source: Int J Mol Sci. 2023 Mar 14;24(6):5548. doi: 10.3390/ijms24065548 (PMC10055992; doi:10.3390/ijms24065548)
Supplement: Supplementary file 1 [file ijms-24-05548-s001.zip › supp figures v2.pdf]

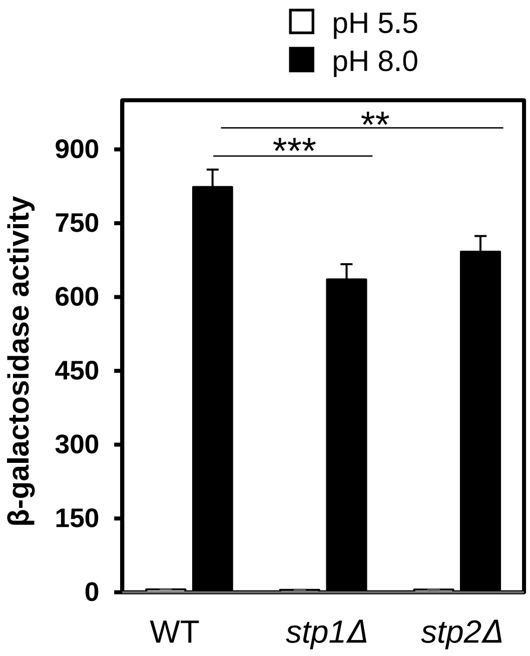

Figure S1. Effect of individual mutations in the *STP1* and *STP2* genes on the expression driven by the full *ENA1* promoter. Plasmid pKC201, bearing the entire *ENA1* promoter, was introduced into strain BY4741 (WT) and its isogenic *stp1* and *stp2* derivatives. Cells were exposed to alkaline pH for 1 h and processed for LacZ activity determination. Data are mean +/- SEM from 3 independent experiments. **, $p<0.01$; ***, $p<0.005$.

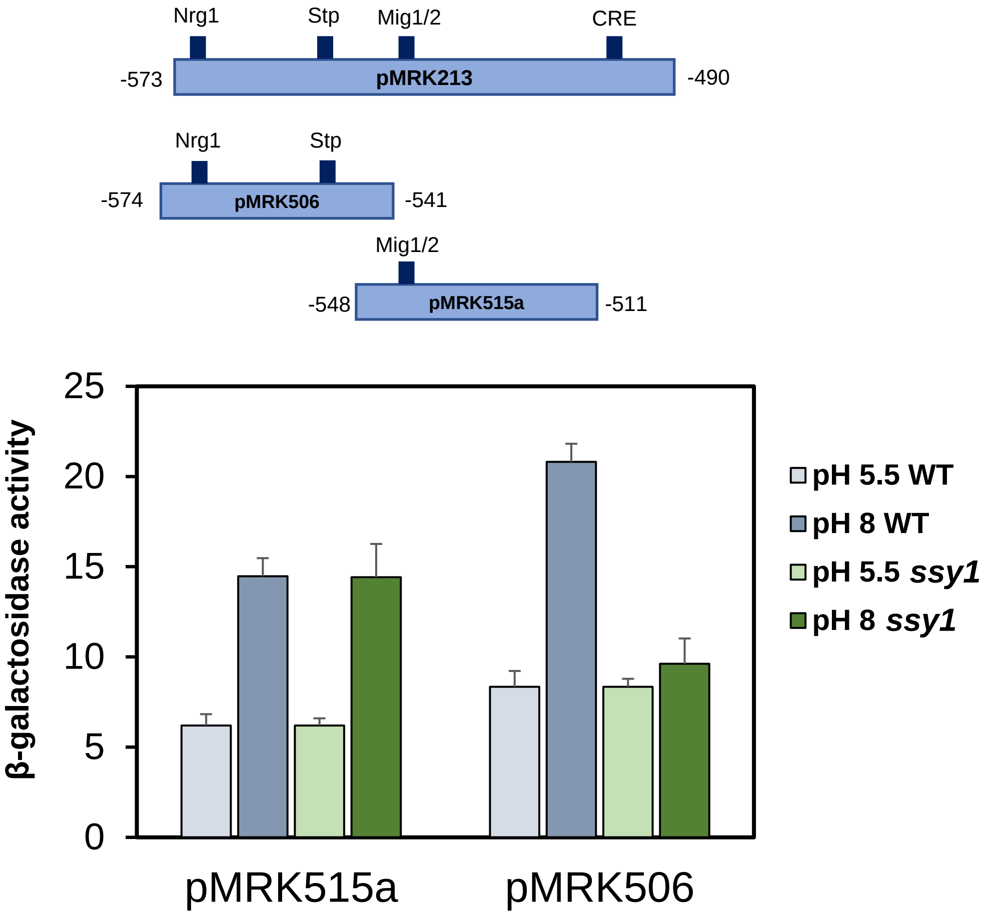

Figure S2.- Functional mapping of the alkaline pH response of the *ENA1* promoter region contained in plasmid pMRK213 in wild type and *ssy1* strains. Cells (23344c background) were transformed with pSLFΔ-178K derived reporters containing the two shorter fragments depicted at the top of the figure and cells were exposed for 1 h to alkaline pH prior determination of β-galactosidase activity. The relevant regulatory sites included in each fragment are depicted. Data are mean ± SEM from 4 independent experiments.

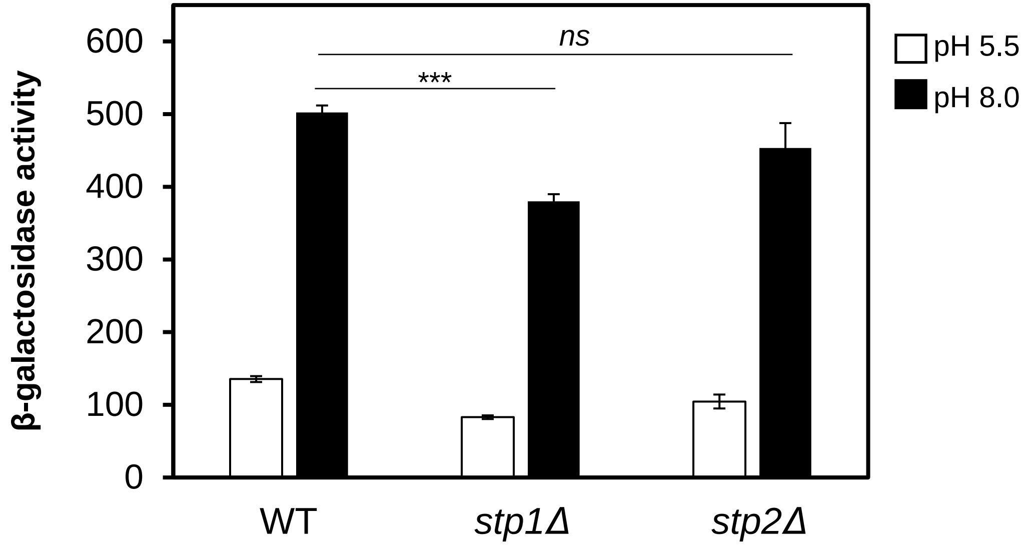

Figure S3.- Effect of the independent *stp1* and *stp2* mutations on the expression from the *HXT2* promoter in the BY4741 background. Cells were grown and processed for β–galactosidase activity determination as in Figure 8. Data are the mean ± SEM from 6 independent cultures. *ns*, $p>0.05$; ***, $p<0.005$.
